# eIF2B as a Target for Viral Evasion of PKR-Mediated Translation Inhibition

**DOI:** 10.1128/mBio.00976-20

**Published:** 2020-07-14

**Authors:** Jennifer Deborah Wuerth, Matthias Habjan, Markus Kainulainen, Besim Berisha, Damien Bertheloot, Giulio Superti-Furga, Andreas Pichlmair, Friedemann Weber

**Affiliations:** a Institute for Virology, FB10-Veterinary Medicine, Justus-Liebig University, Giessen, Germany; b Institute for Virology, Philipps-University Marburg, Marburg, Germany; c Institute of Innate Immunity, University of Bonn, Bonn, Germany; d Innate Immunity Laboratory, Max-Planck Institute of Biochemistry, Munich, Germany; e CeMM Research Center for Molecular Medicine of the Austrian Academy of Sciences, Vienna, Austria; f Center for Physiology and Pharmacology, Medical University of Vienna, Vienna, Austria; g Institute of Virology, Technical University of Munich, School of Medicine, Munich, Germany; h German Center for Infection Research (DZIF), partner sites Giessen and Munich, Germany; The University of Texas Medical Branch at Galveston; University of Colorado School of Medicine

**Keywords:** PKR, phospho-eIF2α, translation inhibition, integrated stress response, viral PKR antagonist, eIF2B, sandfly fever Sicilian phlebovirus, NSs protein

## Abstract

RNA-activated protein kinase (PKR) is a major innate immune factor that senses viral double-stranded RNA (dsRNA) and phosphorylates eukaryotic initiation factor (eIF) 2α. Phosphorylation of the α subunit converts the eIF2αβγ complex into a stoichiometric inhibitor of eukaryotic initiation factor eIF2B, thus halting mRNA translation. To escape this protein synthesis shutoff, viruses have evolved countermechanisms such as dsRNA sequestration, eIF-independent translation by an internal ribosome binding site, degradation of PKR, or dephosphorylation of PKR or of phospho-eIF2α. Here, we report that sandfly fever Sicilian phlebovirus (SFSV) confers such a resistance without interfering with PKR activation or eIF2α phosphorylation. Rather, SFSV expresses a nonstructural protein termed NSs that strongly binds to eIF2B. Although NSs still allows phospho-eIF2α binding to eIF2B, protein synthesis and virus replication are unhindered. Hence, SFSV encodes a unique PKR antagonist that acts by rendering eIF2B resistant to the inhibitory action of bound phospho-eIF2α.

## Abstract (precis)

RNA-activated protein kinase (PKR) is one of the most powerful antiviral defense factors of the mammalian host. PKR acts by phosphorylating mRNA translation initiation factor eIF2α, thereby converting it from a cofactor to an inhibitor of mRNA translation that strongly binds to initiation factor eIF2B. To sustain synthesis of their proteins, viruses are known to counteract this on the level of PKR or eIF2α or by circumventing initiation factor-dependent translation altogether. Here, we report a different PKR escape strategy executed by sandfly fever Sicilian virus (SFSV), a member of the increasingly important group of phleboviruses. We found that the nonstructural protein NSs of SFSV binds to eIF2B and protects it from inactivation by PKR-generated phospho-eIF2α. Protein synthesis is hence maintained and the virus can replicate despite ongoing full-fledged PKR signaling in the infected cells. Thus, SFSV has evolved a unique strategy to escape the powerful antiviral PKR.

## INTRODUCTION

Protein kinase R (PKR) is a major host defense factor against viruses that acts by inhibiting mRNA translation (1). PKR senses viral double-stranded RNA (dsRNA) and phosphorylates Ser51 of the α subunit of eukaryotic initiation factor (eIF) 2. The heterotrimeric eIF2αβγ complex is a GTPase pivotal for initiation of mRNA translation. In its GTP-bound form, the eIF2αβγ complex loads initiator tRNAi-Met onto 40S ribosome subunits. AUG recognition on the mRNA then stimulates GTP hydrolysis, followed by eIF2·GDP release and 60S subunit joining. eIF2·GTP is then recycled by the guanine nucleotide exchange factor eIF2B. Phosphorylation by PKR, however, converts eIF2α from a substrate to a competitive inhibitor of eIF2B, forcing eIF2B into a so-called nonproductive state that halts the translation of mRNAs. To sustain synthesis of their proteins, viruses have therefore evolved escape mechanisms for the PKR-eIF2α signaling cascade, the so-called PKR antagonists. The strategies known so far involve sequestration of dsRNA, circumventing eIFs by means of a special internal ribosome entry site (IRES), PKR sequestration, inhibition of PKR phosphorylation, degradation of PKR, or dephosphorylation of eIF2α (2, 3).

Members of the arthropod-transmitted genus *Phlebovirus* (order *Bunyavirales*, family *Phenuiviridae*) are globally emerging pathogens with significant public health and economic impacts (4). The long-known Rift Valley fever virus (RVFV) can cause encephalitis or hemorrhagic fever in humans and abortion storms and high death rates in ruminants (5). Human infection with the recently emerged severe fever with thrombocytopenia syndrome virus in Asia or the related Heartland virus in North America lead to multiorgan dysfunction with a high case fatality rate (6). Moreover, intermediately virulent phleboviruses such as sandfly fever Sicilian virus (SFSV), Punta Toro virus (PTV), or Toscana virus (TOSV) can cause an incapacitating febrile disease with sudden onset, myalgia, headache, malaise, leukocytopenia, and ocular or gastrointestinal symptoms that may (in the case of TOSV) develop into severe encephalitis (7). SFSV in particular, originally isolated by Albert Sabin after an outbreak of so-called sandfly fever (or “dog disease”) among Allied forces during the invasion of Sicily in 1943 (8), turned out to be one the most widespread and prevalent phleboviruses. SFSV is found in a geographic area from Portugal to India in Eurasia and to Somalia in Africa, with seroprevalences up to 50% in humans and nearly 80% in domestic animals (9–12). While SFSV continues to cause disease in immunologically naive soldiers deployed to areas of endemicity, it becomes increasingly relevant also in travel medicine (9, 12–15). Despite their wide geographical spread, the high risk for exposure, continuing case reports, and their emerging nature, only little is known about SFSV and SFSV-like viruses at the molecular level.

Phleboviruses possess a single-stranded trisegmented RNA genome. The large (L) and medium (M) segments carry the genes for the viral polymerase L or multiple nonstructural proteins and the glycoproteins Gn and Gc, respectively, in negative-sense orientation. In contrast, the small (S) segment uses an antisense configuration to carry the gene for the nonstructural protein NSs in addition to the one for the nucleocapsid protein N.

Replication and transcription of the phlebovirus genome take place in the cytoplasm and can activate PKR (16, 17). For two phleboviruses, RVFV and TOSV, it was previously shown that they express a nonstructural protein (NSs) that triggers proteasomal degradation of PKR (16, 17). However, for the related human pathogen SFSV, although it also encodes an NSs, there is no such PKR degradation, and it remained unclear whether it can escape PKR at all (17, 18). Of note, the NSs proteins of different phleboviruses show only little sequence conservation, which is reflected by the differences in their subcellular localization, host interactomes, and the molecular mechanisms employed to perturb host cell responses (4). Here, we investigated whether and how SFSV, one of the most widespread and prevalent phleboviruses, may be coping with the PKR system. We show that its NSs confers PKR resistance by a unique strategy that targets the downstream factor eIF2B rather than PKR or phospho-eIF2α.

## RESULTS

### PKR escape activity is a trait of several phleboviral NSs proteins.

**FIG 1:**
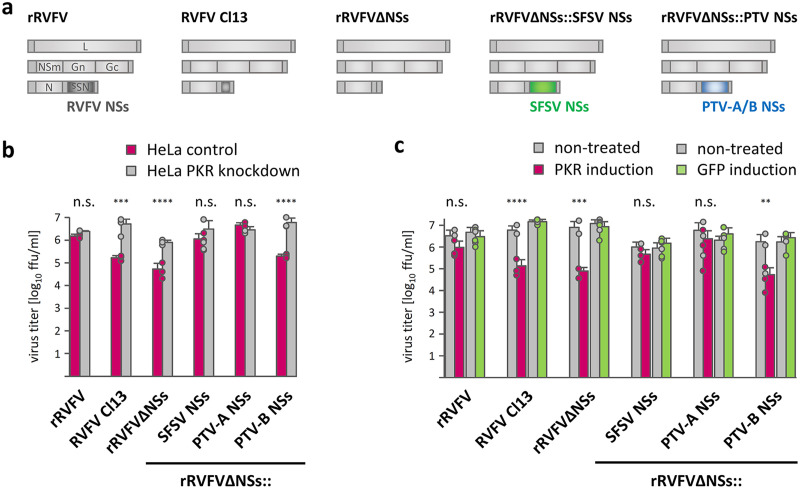
SFSV NSs rescues PKR-sensitive virus replication. (a) Tripartite single-stranded RNA genomes of the virus panel used: wt rRVFV (strain ZH548), natural NSs-deleted RVFV strain clone 13 (Cl13), and recombinant RVFVs expressing no NSs (rRVFVΔNSs) or the NSs genes of SFSV, PTV-A, or PTV-B. (b and c) Replication of the viruses in the absence or presence of PKR. HeLa cells with a stable PKR knockdown and PKR-expressing control cells (b) or doxycycline-induced HEK293 FLP-IN PKR or GFP cells (c) were infected at an MOI of 0.01, and viral titers were determined 24 h later (*n* = 3, mean ± SD). ****, *P* ≤ 0.0001; ***, *P* < 0.001; **, *P* < 0.01; n.s., not significant, *P* > 0.05 (two-way analysis of variance [ANOVA]).

In a first set of experiments addressing potential effects of SFSV NSs on PKR, we employed a recombinant RVFV (rRVFVΔNSs::SFSV NSs), in which the RVFV NSs gene was replaced by the NSs of SFSV (19), along with recombinant wild-type (wt) RVFV and RVFV NSs deletion mutants as controls (Fig. 1a). Here, we also included rRVFV expressing NSs of the PTV phlebovirus strains Adames (PTV-A; virulent) or Balliet (PTV-B; avirulent), but these could not be followed up later due their transcription shutoff activity (see below). The replicative capacity of the various RVFV recombinants was first compared in PKR knockdown versus PKR-expressing control cell lines. As expected from the PKR-destroying activity of RVFV NSs (17, 20), recombinant wild-type RVFV (rRVFV) grew to similar titers in both cell lines, whereas recombinant (rRVFVΔNSs) or natural (RVFV clone 13) NSs deletion mutants exhibited reduced replication in control cells but reached levels comparable to those of rRVFV in PKR knockdown cells (Fig. 1b). The NSs proteins of SFSV as well as of PTV-A also enabled similar growth efficiencies in both cell lines, whereas PTV-B NSs had no such activity. Of note, clone 13 and PTV-B are natural isolates from febrile humans (21, 22), and the NSs protein of PTV-B (but not clone 13) retains type-I interferon (IFN) antagonist function in murine cells (23). We also infected HEK293 FLP-IN cells in which the expression of PKR (or green fluorescent protein [GFP] as control) was induced by doxycycline treatment. Also, both NSs-deficient control viruses as well as the PTV-B NSs recombinant were highly and specifically sensitive to PKR, whereas recombinants containing the NSs genes of SFSV or PTV-A showed only a minor reduction in titers under conditions of PKR induction, similarly to recombinant wild-type RVFV (Fig. 1c). Thus, PKR depletion and overexpression experiments suggest that the NSs proteins of SFSV (and PTV-A) confer PKR resistance.

### PKR escape activity that does not affect PKR signaling.

**FIG 2:**
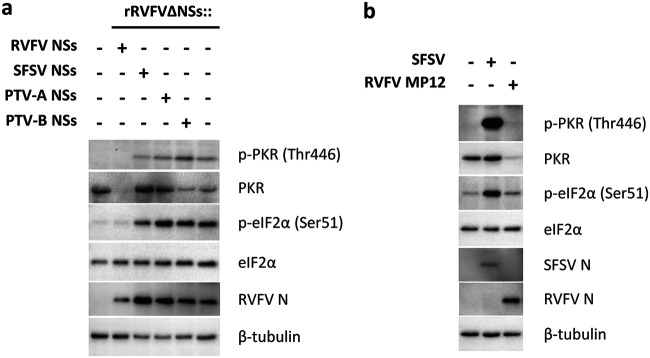
SFSV NSs affects neither PKR activation nor eIF2α phosphorylation. (a) A549 cells were infected with the recombinant RVFVs expressing the various NSs genes presented in Fig. 1 at an MOI of 1 and harvested 8 hpi for immunoblot analysis (representative of 2 experiments). Staining of RVFV N served as marker for viral infection common to all recombinant viruses. (b) A549 cells infected with SFSV or RVFV strain MP12 (MOI 1) and harvested 12 hpi were analyzed by immunoblotting (representative of 5 experiments).

We then investigated the influence of the phleboviral NSs proteins on the PKR signaling pathway. Infection with the recombinant viruses (Fig. 2a) confirmed that the NSs of RVFV, but not of SFSV or PTV, reduces PKR levels (17, 18, 20). Consequently, autophosphorylated PKR, an indicator of PKR activity, was undetectable in the presence of RVFV NSs. Curiously, however, when cells were infected with viruses expressing SFSV or PTV NSs, phosphorylation of both PKR and its substrate eIF2α was upregulated similar to that in the NSs deletion virus rRVFVΔNSs (Fig. 2a). Time course analyses showed that PKR and eIF2α phosphorylation persisted despite the presence of SFSV or PTV NSs (see Fig. S1 in the supplemental material). In line with this, parental SFSV also triggered PKR and eIF2α phosphorylation, unlike the PKR-destroying RVFV (Fig. 2b). Thus, on one hand, the NSs proteins of SFSV and PTV are required to counter the antiviral activity of PKR (Fig. 1), indicating a PKR escape phenotype. On the other hand, however, these NSs proteins seem to act in a manner that is different from that of prototypical PKR antagonists, as they do not interfere with the PKR-eIF2α phosphorylation axis.

10.1128/mBio.00976-20.1 FIG S1 Kinetics of PKR and eIF2α phosphorylation. A549 cells were infected with a recombinant wild-type RVFV (rRVFV) (a), naturally NSs-deleted strain clone 13 (Cl13) (b), or recombinant viruses containing the NSs genes of SFSV (c), PTV-A (d), PTV-B (e), or NSs-deficient RVFV (f) and harvested at the indicated time points for immunoblot analysis (representative of 2 experiments). Lysates of mock and rRVFVΔNSs-infected cells run on all gels were both harvested 16 hpi. Staining of RVFV N protein served as marker for infection. Note that levels of activated PKR can decrease over time, as described previously (59). Download FIG S1, TIF file, 1.8 MB. Copyright © 2020 Wuerth et al. 2020 Wuerth et al. This content is distributed under the terms of the Creative Commons Attribution 4.0 International license.

### SFSV NSs enhances eIF2-dependent translation.

**FIG 3:**
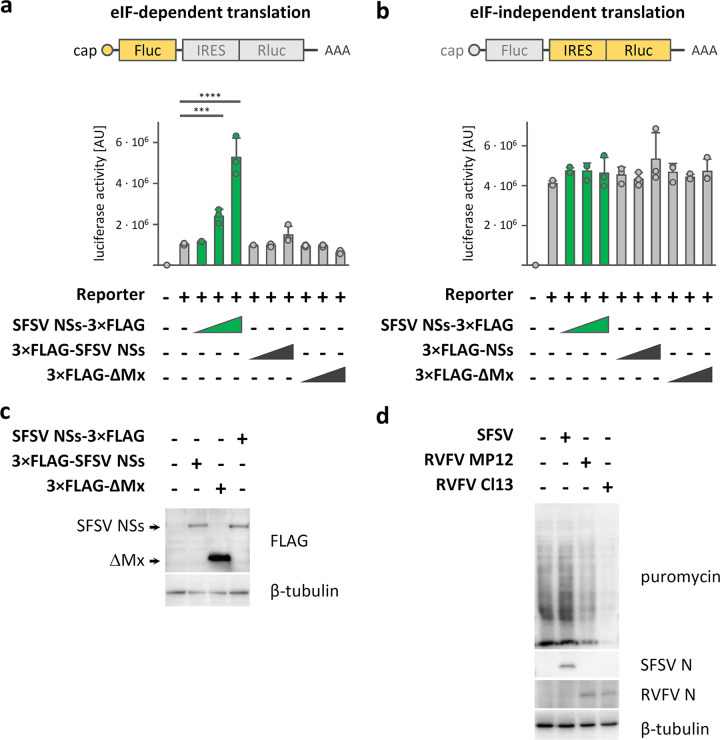
Effect of SFSV NSs on mRNA translation. (a and b) Influence of SFSV NSs on a bicistronic reporter system with an upstream canonical (i.e., eIF-dependent) firefly luciferase ORF and a downstream *Renilla* luciferase ORF initiated from the eIF-independent IRES_CrPV_. HEK293 cells were transfected with the bicistronic reporter plasmid and expression plasmids for SFSV NSs containing either a C- or N-terminal 3×FLAG tag (SFSV NSs-3×FLAG and 3×FLAG-NSs SFSV, respectively) or for an irrelevant negative control (3×FLAG-ΔMx). Lysates were assayed for eIF2-dependent firefly (Fluc) (a) and IRES-dependent *Renilla* luciferase (Rluc) (b) activities (arbitrary units [AU]). Shown are data from 1 of 3 independent experiments. Each of these was conducted with three technical (i.e., parallel) replicates. Bars show means ± SDs. ****, *P* ≤ 0.0001; ***, *P* < 0.001 (one-way ANOVA, corrected for multiple comparisons using Dunnett test). For the Rluc data, none of the tests reached the significance level. (c) C- and N-terminally tagged SFSV NSs (SFSV NSs-3×FLAG and 3×FLAG-NSs SFSV, respectively) were transiently expressed in HEK293 cells, and expression levels were analyzed by immunoblotting (representative of 3 experiments). (d) A549 cells were infected with SFSV, MP12, or clone 13 (MOI, 1), and currently translated proteins were labeled by the addition of puromycin 12 h postinfection, followed by subsequent immunoblot analysis (representative of 4 experiments). Staining of viral N proteins served as marker for infection. Data are representative and from 1 of 3 independent experiments each with 3 technical replicates (a and b), from 1 of 3 independent experiments (c), or from 1 of 4 independent experiments (d).

Transfection of DNA plasmids is known to activate PKR (24). Hence, we aimed to assess the impact of SFSV NSs on cellular mRNA translation by using a transiently transfected luciferase reporter system. PTV NSs could not be included in these and further experiments as it impairs general host transcription (18) (data not shown). The luciferase reporter system encodes a bicistronic mRNA in which translation of the upstream firefly luciferase open reading frame (ORF) is canonically initiated from the 5′ cap (and hence requires eIF2), whereas translation of the downstream *Renilla* luciferase ORF is initiated from the cricket paralysis virus IRES (IRES_CrPV_) that does not require any eIFs (25). Cotransfection of SFSV NSs with the bicistronic reporter construct amplified firefly luciferase activity, i.e., eIF-dependent gene expression, in a dose-dependent manner (Fig. 3a), whereas it had no detectable effect on eIF-independent *Renilla* luciferase activity (Fig. 3b). Of note, the boost of firefly luciferase activity was observable only for C-terminally epitope-tagged NSs (SFSV NSs-3×FLAG), but not for the N-terminally tagged variant (3×FLAG-SFSV NSs) or the inert negative control (3×FLAG-ΔMx) (Fig. 3a). Both the C-terminally and the N-terminally tagged NSs proteins were, however, expressed at comparable levels (Fig. 3c) and exhibited the previously reported (19) inhibitory activity toward transcriptional induction of type I interferon (see Fig. S2a and c). Consistent with the results from the bicistronic luciferase assay, C-terminally tagged SFSV NSs also boosted an eIF-dependent SV40 luciferase reporter (Fig. S2b). Furthermore, puromycin labeling of *de novo* synthesized proteins showed that mRNA translation was comparable to that in noninfected cells during wt SFSV infection, whereas infection with the PKR-activating NSs mutant clone 13 (Fig. S1) led to a shutdown of protein synthesis (Fig. 3d). Thus, unlike other previously characterized viral PKR antagonists, SFSV NSs enables both viral replication and canonical eIF-dependent mRNA translation without blocking PKR activation or affecting the phosphorylation state of eIF2α.

10.1128/mBio.00976-20.2 FIG S2 Position of the epitope tag does not impair the ability of SFSV NSs to inhibit IFN induction. C- or N-terminally tagged SFSV NSs was coexpressed in HEK293 cells together with MAVS and firefly (Fluc) and *Renilla* (Rluc) luciferases under the control of the *Ifnb1* and SV40 promoters, respectively. (a and b) Raw counts for firefly and *Renilla* luciferase activities (*Ifnb1*- and SV40-dependent promoter activities). (c) Normalized *Ifnb1* activities. *n* = 3, mean ± SD, representative of 4 independent experiments. ****, *P* ≤ 0.0001; ***, *P* < 0.001; *, *P* < 0.05 (one-way ANOVA, corrected for multiple comparisons using Dunnett test). Download FIG S2, TIF file, 0.6 MB. Copyright © 2020 Wuerth et al. 2020 Wuerth et al. This content is distributed under the terms of the Creative Commons Attribution 4.0 International license.

### SFSV NSs interacts with the eIF2B complex.

**FIG 4:**
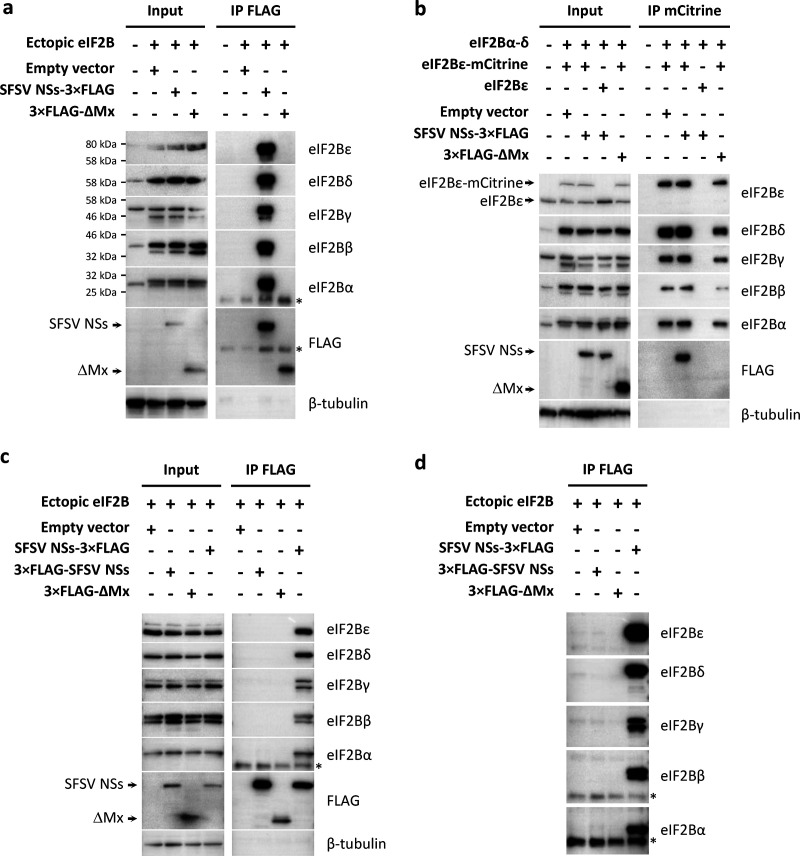
Interaction of SFSV NSs with the eIF2B complex. Coimmunoprecipitation experiments. HEK293 cells were transiently transfected with expression plasmids for all eIF2B subunits as well as 3×FLAG-tagged SFSV NSs variants or the unrelated control 3×FLAG-ΔMx. Proteins in the cell lysates were precipitated via specific tags and analyzed by immunoblotting with the indicated antibodies. (a) Overexpression of eIF2B subunits along with C-terminally 3×FLAG-tagged NSs (SFSV NSs-3×FLAG). Immunoprecipitation was performed with anti-FLAG antibody. (b) mCitrine-HA-tagged eIF2Bε (eIF2Bε-mCitrine-HA) was expressed along with eIF2B subunits α, β, γ, and δ as well as SFSV NSs-3×FLAG. Untagged eIF2Bε served as control for unspecific binding. The eIF2B complex was precipitated using an mCitrine-binding matrix. (c) Overexpression of untagged eIF2B subunits along with C- or N-terminally 3×FLAG-tagged NSs (SFSV NSs-3×FLAG and 3×FLAG-NSs SFSV, respectively). Immunoprecipitation was performed with anti-FLAG antibody. (d) Immunoprecipitated proteins from the experiment shown in panel c after prolonged exposure. Shown data are representatives of 3 (a, c, and d) or 5 (b) experiments. *, light chain of the IP antibody.

SFSV NSs seems to protect protein synthesis by neutralizing antiviral PKR action downstream of eIF2α. Ser51 phosphorylation of eIF2α is known to convert the eIF2αβγ complex from a substrate into an inhibitor of its guanine nucleotide exchange factor eIF2B (26). Strikingly, our previous mass spectrometry-based approach to map the host interactome of SFSV NSs returned all five subunits of the eIF2B complex as the highest scoring candidate interactors (see Fig. S3) (27). To verify the interaction, we overexpressed the eIF2B subunits from cDNA plasmids together with C-terminally 3×FLAG-tagged SFSV NSs and performed immunoprecipitations for NSs or eIF2B with antibodies to epitope tags. Immunoprecipitation of FLAG-tagged NSs, but not the FLAG-tagged control protein (ΔMx), coprecipitated all five eIF2B subunits in a highly reproducible manner (Fig. 4a). Vice versa, when using an expression construct for epitope-tagged eIF2Bε-mCitrine-hemagglutinin (HA), we were able to pull down the entire eIF2B complex via the mCitrine tag and also SFSV NSs (Fig. 4b). As a specificity control, we expressed untagged eIF2Bε instead of eIF2Bε-mCitrine-HA and observed no such precipitations, as expected. Furthermore, SFSV NSs also enabled specific coprecipitation of endogenous eIF2B from both A549 and HEK293 cells (Fig. S4a and data not shown), and superinfection with a PKR-activating NSs-deficient RVFV did not affect its interaction with eIF2B (Fig. S4b). Finally, only the translation-rescuing SFSV NSs with the C-terminal FLAG tag (SFSV NSs-3×FLAG) but not the inactive N-terminally tagged version (3×FLAG-SFSV NSs) interacted with eIF2B (Fig. 4c and d). This result correlates with the functional data from the bicistronic reporter system (Fig. 3), suggesting that SFSV NSs boosts eIF-dependent translation and enables PKR-sensitive virus replication by acting directly on eIF2B.

10.1128/mBio.00976-20.3 FIG S3 Candidate host interactors of SFSV NSs. STRING analysis of the network of candidate host interactors of SFSV NSs (27). eIF2B subunits are highlighted in orange. Download FIG S3, TIF file, 1.7 MB. Copyright © 2020 Wuerth et al. 2020 Wuerth et al. This content is distributed under the terms of the Creative Commons Attribution 4.0 International license.

10.1128/mBio.00976-20.4 FIG S4 SFSV NSs acts on eIF2B. (a) Coimmunoprecipitation of endogenous eIF2B from A549 cells with SFSV NSs-3×FLAG (representative of 3 experiments). (b) Coimmunoprecipitation of overexpressed eIF2B via NSs from HEK293 cells additionally infected with rRVFVΔNSs::Ren or left untreated (representative of 3 experiments). *, light chain of the IP antibody; **, heavy chain of the IP antibody. Download FIG S4, TIF file, 1.4 MB. Copyright © 2020 Wuerth et al. 2020 Wuerth et al. This content is distributed under the terms of the Creative Commons Attribution 4.0 International license.

Further attempts at identifying the eIF2B subunit(s) targeted by SFSV NSs using Far-Western blotting (employing cells that transiently expressed NSs-3×FLAG as bait and individual, bacterially expressed nondenatured eIF2B subunits as prey) did not reveal any interaction (data not shown). Moreover, bacterially produced SFSV NSs lost its ability to interact with cellular eIF2B in coimmunoprecipitation experiments, precluding binding studies with NSs produced in an eIF2B-free background (data not shown).

### Established mechanisms of cellular eIF2B modulation are not applicable to SFSV NSs.

**FIG 5:**
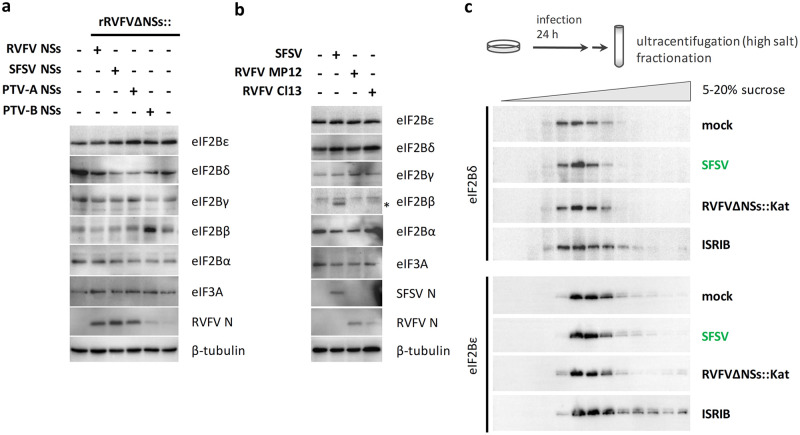
SFSV NSs and established mechanisms of eIF2B regulation. (a and b) Monitoring expression levels of eIF2B subunits in the presence of NSs. A549 cells were either infected with the indicated recombinant viruses (MOI, 1) and lysed 16 hpi (a) or infected with parental viruses SFSV, RVFV MP12, and Cl13 (MOI, 1) and lysed 12 hpi (b) and analyzed by immunoblotting. Staining of viral N proteins served as marker for infection. *, leftover SFSV N signal, for which the blot was probed before detecting eIF2B beta. (c) Monitoring eIF2B decamer formation. HEK293 cells were infected with SFSV or NSs-deficient RVFV strain rRVFVΔNSs::Katushka (rRVFVΔNSs::Kat) or treated with ISRIB. rRVFVΔNSs::Kat served as negative control for infection-induced but NSs-independent effects on eIF2B stoichiometry, whereas ISRIB was included as positive control for eIF2B decamerization. Cell lysates were fractionated via 5% to 20% sucrose gradients, and fractions were analyzed for a shift of eIF2B subunits toward fractions of higher density by immunoblotting. Shown data are representatives of 2 (a) or 3 (b and c) experiments.

Phosphorylation of eIF2α is not only mediated by PKR but also by other kinases of the so-called integrated stress response (ISR) (28). Besides virus infection, the ISR can also be activated by compounds such as arsenite. Due to its low cellular levels and tight regulation, eIF2B is considered the central hub of translation regulation by the ISR (26). Cancer cells, for example, elevate eIF2B expression to satisfy their demand for increased protein synthesis (29). Moreover, the stabilization of the decameric form of eIF2B (consisting of two copies of each of the five subunits), a process facilitated by the small molecule ISRIB (integrated stress response inhibitor), enables translation despite the presence of phospho-eIF2α (30–32). However, when testing for these mechanisms, we did not find SFSV NSs to elevate eIF2B levels under ectopic expression or infection (Fig. 5a and b and data not shown). Moreover, after sucrose gradient ultracentrifugation of cell lysates, a shift of eIF2Bδ and eIF2Bε toward high-density fractions that is indicative of decamer formation was only detected for the ISRIB control and not for NSs-expressing SFSV or the ISR-activating rRVFVΔNSs::Kat virus (Fig. 5c and data not shown). Thus, despite strongly binding to eIF2B, SFSV NSs seems not to act by any of the established phospho-eIF2α bypass mechanisms.

### SFSV NSs does not interfere with binding of phospho-eIF2α to eIF2B.

**FIG 6:**
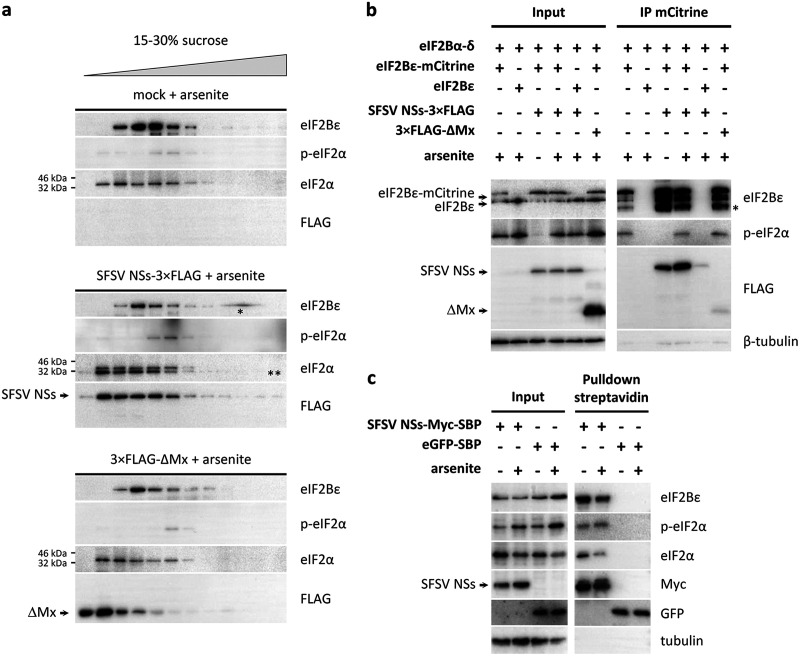
SFSV NSs allows binding of phospho-eIF2α to eIF2B. (a) Cofractionation experiments. HEK293 cells were transfected with expression plasmids for SFSV NSs-3×FLAG or 3×FLAG-ΔMx (negative control) or left untransfected. Half of the cells was stimulated with arsenite to induce eIF2α phosphorylation prior to lysis (see Fig. S5 in the supplemental material for results with the unstimulated cells). Sucrose gradient ultracentrifugation was performed, and fractions were analyzed by immunoblotting for phospho-eIF2α with eIF2B. *, gel artifact; **, remaining signal for SFSV NSs-3×FLAG (lower band) from previous FLAG antibody staining due to incomplete stripping of the anti-FLAG antibody. (b and c) Coprecipitation experiments. HEK293 cells were transfected with expression plasmids, treated with arsenite to induce eIF2α phosphorylation, and lysed, and protein complexes were precipitated via specific tags and analyzed by immunoblotting. (b) eIF2B pulldown. eIF2Bε-mCitrine, SFSV NSs, and ΔMx were expressed, and eIF2B was precipitated via the mCitrine tag. *, cleavage or partial degradation product of eIF2Bε. (c) NSs pulldown. SFSV NSs-Myc-SBP and eGFP-SBP were expressed, and NSs was precipitated via the SBP tag. Shown data are representatives of 2 (a), 5 (b), or 3 (c) experiments.

Comparative cryo-electron microscopy (cryo-EM) analyses have recently elucidated that nonphosphorylated and phosphorylated eIF2 bind to distinct sites on eIF2B (33–36). Binding of nonphosphorylated eIF2α triggers the so-called productive mode of eIF2B in which eIF2·GTP is recycled and translation of mRNAs enabled. In contrast, phospho-eIF2α is structurally rearranged and consequently excluded from the productive binding site of eIF2B. Instead, it associates with eIF2B in a nonproductive binding mode, through which access of nonphosphorylated eIF2 to eIF2B is blocked and hence eIF2B activity abrogated, halting translation. To test the influence of SFSV NSs on the binding of phospho-eIF2α to eIF2B, we performed cofractionation as well as coimmunoprecipitation experiments. In the cofractionation experiments, lysates from cells that were treated with the ISR activator arsenite were separated by sucrose gradient ultracentrifugation, and the fractions were analyzed by immunoblotting. In lysates from control cells, the phospho-eIF2α peak was found to overlap that for fractions containing eIF2Bε, suggesting complex formation (Fig. 6a and Fig. S5). However, expression of SFSV NSs did not shift the phospho-eIF2α peak toward lower-density fractions, as would have been expected if it interfered with the binding of phospho-eIF2α to eIF2B. Rather, phospho-eIF2α was exclusively recovered in the fractions containing eIF2Bε, similar to the situation in untransfected or control (ΔMx) transfected cells (Fig. 6a).

10.1128/mBio.00976-20.5 FIG S5 SFSV NSs allows binding of phospho-eIF2α to eIF2B. Unstimulated controls for Fig. 6b: sucrose gradient ultracentrifugation under ectopic expression of SFSV NSs-3×FLAG or 3×FLAG-ΔMx in HEK293 cells without subsequent stimulation (representative of 2 experiments). *, remaining signal (lower band) from previous FLAG antibody staining for NSs-FLAG. Download FIG S5, TIF file, 1.2 MB. Copyright © 2020 Wuerth et al. 2020 Wuerth et al. This content is distributed under the terms of the Creative Commons Attribution 4.0 International license.

For the coimmunoprecipitations, two different approaches were chosen. First, we coexpressed the eIF2Bε-mCitrine-HA-containing eIF2B complex with SFSV NSs, stimulated eIF2α phosphorylation with arsenite, and subsequently immunoprecipitated eIF2B via the mCitrine moiety. Phospho-eIF2α specifically and reproducibly coprecipitated with eIF2B as expected (Fig. 6b). eIF2α phosphorylation had no effect on the amount of SFSV NSs binding to eIF2B. Vice versa, the presence of SFSV NSs also did not reduce the signal of coprecipitated phospho-eIF2α. To further support these findings, we directly investigated whether phospho-eIF2α is attached to NSs-precipitated eIF2B. To this aim, we constructed an expression plasmid for NSs (NSs-Myc-streptavidin-binding peptide [SBP]) that was C-terminally tagged with Myc (for immunoblot detection) and streptavidin-binding peptide (for precipitation with streptavidin-coated beads). eGFP-SBP served as negative control. Cells were transfected with these cDNA constructs, the ISR was stimulated with arsenite, and the lysates were subjected to streptavidin-mediated precipitation. As observed previously with the NSs-3×FLAG construct, NSs-Myc-SBP coprecipitated the endogenous eIF2B (represented by the eIF2Bε subunit), whereas eGFP-SBP did not (Fig. 6c). Importantly, endogenous phospho-eIF2α also specifically coprecipitated along with the NSs-eIF2B complex, demonstrating that NSs does not impede the binding of phospho-eIF2α to eIF2B. Thus, the results from the fractionation and coprecipitation experiments argue for a model in which SFSV NSs is binding to the eIF2B-phospho-eIF2 complex. Because, on the other hand, SFSV NSs enables virus replication despite the presence of phospho-PKR and phospho-eIF2α, our data suggest that it renders eIF2B resistant to inhibition by bound phospho-eIF2.

## DISCUSSION

Phleboviruses are gaining increased attention as agents of emerging zoonoses (4, 7, 37). As RNA viruses infecting vertebrates, they have to cope with the broadly antiviral PKR system, and the virulent phleboviruses RVFV and TOSV rapidly destroy PKR itself as a countermeasure (17). SFSV, one of the most widespread phleboviruses, is a more moderate pathogen and, as we show here, inhibits the PKR-mediated translation block by targeting eIF2B. All phleboviruses investigated so far also inhibit induction of the antiviral interferons (IFN-α/β) (7, 38, 39). Interestingly, virulent RVFV and TOSV do this by NSs-mediated destruction of key host factors, whereas SFSV NSs obstructs the DNA-binding domain of transcription factor IRF3, blocking its access to the IFN promoter (19). Thus, there seems to be a correlation between the mode of NSs action against host defenses (rapid and enzymatic versus slowly building up and stoichiometric) and the respective virulence level of the particular phlebovirus, observed not only in humans but also in an outbred mouse model (23).

**FIG 7:**
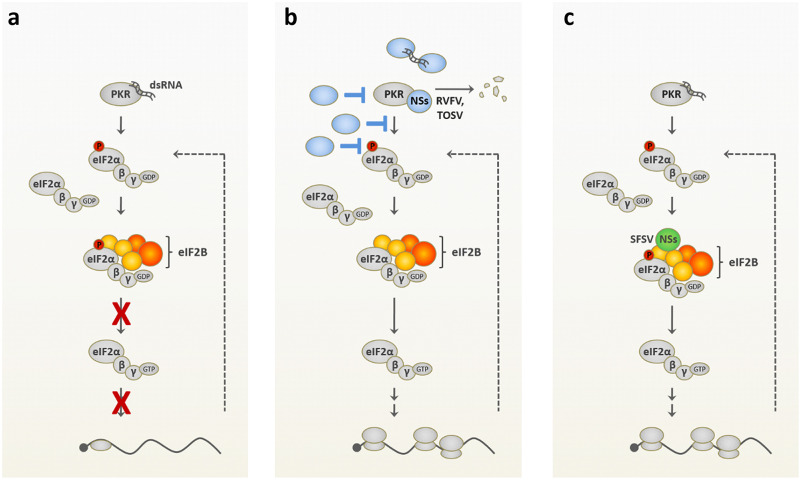
PKR-mediated shutdown of translation and antagonistic strategies employed by viral proteins. (a) Shutdown of translation initiation mediated by PKR: in response to recognition of double-stranded RNA (dsRNA), PKR phosphorylates eIF2αSer51, resulting in nonproductive binding of the latter to eIF2B and, consequently, global inhibition of translation. Due to limiting concentrations of eIF2B compared to that of eIF2, partial eIF2α phosphorylation is sufficient to shutdown protein synthesis. (b) Previously reported viral strategies for PKR antagonism: viral proteins (blue) sequester viral RNA or affect PKR levels (such as NSs proteins of phleboviruses RVFV and TOSV), PKR activation, or the phosphorylation state of eIF2α. (c) Mechanism used by SFSV NSs: while PKR activation, PKR phosphorylation, and eIF2α phosphorylation occur in response to the virus infection and even the binding of p-eIF2α to eIF2B occurs, SFSV NSs enables cap-dependent translation by targeting eIF2B in a way that somehow neutralizes the nonproductive mode of eIF2B imposed by p-eIF2α binding.

Viruses have evolved a plethora of measures to avoid translation inhibition by the antiviral PKR system (Fig. 7a and b). The destruction of PKR by RVFV and TOSV NSs proteins was mentioned above. At the upper end of the signaling chain, PKR-triggering dsRNA is sequestered by the influenza A NS1 protein and the vaccinia virus E3L, among others (40). dsRNA-induced PKR autophosphorylation is inhibited by the Kaposi’s sarcoma herpesvirus vIRF2 (41), vaccinia virus K3L acts as a PKR pseudosubstrate (42), and herpes simplex virus ICP34.5 recruits a protein phosphatase to revert eIF2α phosphorylation (43). Besides these examples, all viral strategies reported so far act on the level of PKR, affect phosphorylation of its substrate eIF2α (2, 3), or circumvent eIF-dependent translation with a special IRES that directly binds to the 40S ribosome (25). Although, mechanistically, viruses may not necessarily target PKR itself, they nonetheless neutralize its negative effect on viral replication by a variety of other means, i.e., biologically, they encode a PKR escape activity. As shown by our knockdown and overexpression experiments, the NSs of SFSV only offers a growth advantage when PKR is present, i.e., phenotypically, it is a PKR antagonist. By targeting eIF2B, however, it employs a distinct and apparently novel PKR escape mechanism (Fig. 7c). There are several modes of cellular eIF2B regulation that can relieve the negative influence of phosphorylated eIF2α, most prominently, upregulation of eIF2B subunits and eIF2B decamerization, but none of these apply to SFSV NSs and NSs did not displace phospho-eIF2α from eIF2B. Thus, NSs can enable eIF2-dependent protein synthesis in the presence of activated PKR by a novel mechanism that protects eIF2B from the nonproductive mode that is normally imposed by phospho-eIF2α.

It remains to be shown where on the eIF2B complex NSs is binding and how NSs manages to force phospho-eIF2-bound eIF2B into the productive mode. One possibility would be that SFSV NSs enables binding of phospho-eIF2 to the productive site that is normally occupied by nonphosphorylated eIF2. However, since phospho-eIF2 is excluded from the productive binding site due to electrostatic repulsion by the eIF2Bβ subunit (35), this possibility appears unlikely. Alternatively, due to its complex architecture, eIF2B offers potential for manifold posttranslational modifications. For instance, Ser539 of the catalytic eIF2Bε subunit is a regulatory site for enzymatic eIF2B activity (44). Although we did not observe differential eIF2Bε Ser539 phosphorylation in the presence of SFSV NSs when using commercial antibodies (data not shown), other posttranslational modifications cannot be ruled out. Moreover, in addition to its known binding sites for phosphorylated and nonphosphorylated eIF2, GDP, and GTP, eIF2B possesses a significant amount of further surface area without an assigned physiological function. It could thus be envisioned that NSs acts as mimic of an as-yet-unidentified host protein with eIF2B regulatory activity. Further elucidation of the supercomplex of eIF2B, phosphorylated eIF2α, and SFSV NSs might aid the ongoing efforts to understand the intricate regulation of eIF2B.

## MATERIALS AND METHODS

### Cell culture.

A549, BHK-21, HEK293, HEK293T, Vero B4, and Vero E6 cells were cultured in Dulbecco’s minimal essential medium (DMEM) and CCM34 medium (DMEM with addition of 17.8 mg/liter l-alanine, 0.7 g/liter glycine, 75 mg/liter l-glutamic acid, 25 mg/liter l-proline, 0.1 mg/liter biotin, 25 mg/liter hypoxanthine, and 3.7 g/liter sodium bicarbonate) supplemented with 10% fetal calf serum (FCS), 100 U/ml penicillin, and 100 μg/ml streptomycin. HeLa PKR knockdown and control cells, generated by stable transfection with the pSUPER vector encoding a short hairpin RNA against the PKR gene or with the empty pSUPER, respectively (kindly obtained from Charles Samuel, UC Santa Barbara) (45), were maintained in full DMEM or CCM34 additionally supplemented with 2 μg/ml puromycin. HEK293 FLP-IN T Rex cells with inducible expression of PKR or GFP were as described previously (17) or obtained from Ju-Tao Guo (Baruch S. Blumberg Institute) (46) and maintained in full DMEM or CCM34 medium additionally supplemented with 50 μg/ml hygromycin and 5 μg/ml blasticidin, respectively. All cell lines were routinely tested for mycoplasma contamination.

### Viruses.

Previously described recombinant RVFV strains rZH548, rZH548ΔNSs, rZH548ΔNSs::NSsSFSV, rZH548ΔNSs::NSsPTV-A, rZH548ΔNSs::PTV-B, and rZH548ΔNSs::NSsSFSV-CTAP (17, 19, 47) were propagated in Vero E6 cells under biosafely level 3 (BSL3) conditions. The prototype Sabin strain of SFSV was obtained from the World Reference Center for Emerging Viruses and Arboviruses (WRCEVA) and propagated in Vero B4 cells. rZH548ΔNSs::Katushka (48) was also propagated in Vero E6 cells, whereas rZH548ΔNSs::Ren (49) as well as attenuated RVFV strains MP12 and clone 13 were propagated in BHK-21 cells. All viruses were titrated on Vero E6 cells under an overlay of 0.6% Avicel (FMC BioPolymer) (50), and plaques were visualized via crystal violet staining. Virus stocks were routinely tested for mycoplasma contamination.

For infection, viruses were diluted to the desired multiplicity of infection (MOI) in serum-free medium and incubated with the cells for 1 h at 37°C, after which, the inoculate was replaced by full cell culture medium.

### Plasmids.

Expression constructs for 3×FLAG-tagged NSs of RVFV and SFSV (GenBank EF201822.1) as well as 3×FLAG-ΔMx were described previously (19, 51). C-terminally tagged SFSV NSs was generated by amplification of the ORF with specific primers, of which, the reverse primer contained the 3×FLAG or Myc-SBP tag sequence, restriction with BamHI and XhoI, and subsequent ligation-dependent cloning into pI.18 (kindly provided by Jim Robertson, National Institute for Biological Standards and Control, Hertfordshire, UK) and pcDNA3.1 (Invitrogen). Coding sequences for eIF2B subunits *eIF2B1* (NG_015862.1), *eIF2B2-B10* (NG_013333.1), *eIF2B3* (NG_015864.1), and *eIF2B4* (NG_009305.1) were amplified from pDONR223 constructs (kindly provided by BIOSS Centre for Biological Signaling Studies, University of Freiburg, Germany), inserted into pcDNA3.1/V5-His-TOPO via TA cloning, and finally subcloned into pcDNA3 via the HindIII and NotI restriction sites. The *eIF2B5* ORF (NM_003907.2) was amplified and subcloned from pRevTRE2-hIF2Bε-GFP (52) (kind gift of Dirk Görlich, Max Planck Institute for Biophysical Chemistry, Germany) and additionally supplemented with an mCitrine-HA cassette using the NotI and ApaI sites. Primer and insert sequences are available upon request. All expression constructs were confirmed by Sanger sequencing with primers covering the respective inserts and multiple-cloning sites.

Translation reporter pFR_CrPV_xb, constructed by Philipp Sharp (53), was obtained from Addgene (plasmid 11509). Subsequently, the HSV-TK promoter was replaced with an SV40 promoter by directional cloning using the BglII and HindIII restriction sites. Firefly reporter construct p-125Luc was kindly donated by Takashi Fujita (54); pGL3-Control and pRL-SV40 were purchased from Promega.

### Replication assays.

HeLa cells were seeded into 6-well plates and infected with recombinant viruses (MOI, 0.01). HEK293 FLP-IN T Rex cells were seeded into 6-well plates, induced with 2 μg/ml doxycycline (Sigma) for 24 h, and infected with recombinant viruses (MOI 0.01) under continuing induction. Culture supernatants were harvested 24 h postinfection (hpi), and virus titers were determined by immunoplaque assay as follows: BHK-21 cells were infected with serial dilutions of cell culture supernatant and incubated under an overlay of 0.6% Avicel (FMC BioPolymer) (50) for 24 h prior to fixation and permeabilization. After staining with polyclonal mouse ascites fluid raised against recombinant RVFV N (kindly provided by Michèle Bouloy, Institut Pasteur Paris, France) (55) and horseradish peroxidase (HRP)-coupled secondary antibody, plaques were visualized using TrueBlue peroxidase substrate (KPL) and titers were calculated.

### Immunoblot analysis.

Protein samples were run on 12% or 15% acrylamide gels and transferred to polyvinylidene fluoride (PVDF) membranes (Millipore) via semidry blotting. After blocking in Tris-buffered saline (TBS) with 5% bovine serum albumin (BSA) or milk powder, primary antibody staining was performed for 1 h at room temperature or overnight at 4°C. Membranes were washed in TBS-0.1% Tween 20, stained with secondary antibodies for 45 min, and washed again in TBS-0.1% Tween 20 and once in TBS. Finally, membranes were developed with a SuperSignal West Femto kit (Pierce), and bands were visualized using a ChemiDoc imaging system and Image Lab software (Bio-Rad). For kinetic analysis after infection with the recombinant virus panel, proteins were blotted onto nitrocellulose membranes (Whatman Protran) and stained as described above, and bands were detected using an Odyssey imaging system (LI-COR).

Primary antibodies were used as follows: β-actin (1:1,000, number [no.] 3700; Cell Signaling); eIF2α (1:1,000, no. 2103; Cell Signaling); phospho (p)-eIF2α (1:500, no. 3597 [Cell Signaling] and 1:1,000, no. 44728G [Invitrogen]); eIF2Bα (sc-98323 [Santa Cruz Biotechnology] and 18010-1-AP [Proteintech], both 1:1,000); eIF2Bβ (1:1,000, sc-100729; Santa Cruz Biotechnology); eIF2Bγ (1:1,000, sc-137248; Santa Cruz Biotechnology); eIF2Bδ (1:500, sc-271795; Santa Cruz Biotechnology); eIF2Bε (1:1,000, sc-55558; Santa Cruz Biotechnology); eIF3A (1:1,000, no. 3411; Cell Signaling); FLAG M2 (1:2,000, F3165; Sigma); GFP (1:1,000, 3h9; Chromotek); HA (1:1,000, no. 901515; BioLegend); Myc (1:1,000, M4439; Sigma); PKR (1:1,000, no. 610764; BD Transduction Laboratories); p-PKR (1:1,000, ab32036; Abcam); puromycin (1:1,000, EQ0001; Kerafast); tubulin (1:2,500, ab6046; Abcam); SFSV N (mouse immune ascites fluid, provided by WRCEVA, 1:1,000); RVFV N (rabbit hyperimmune serum, kindly provided by Alenjandro Brun, 1:1,000). Secondary antibodies comprised anti-mouse (0031430 and 1892913), anti-rabbit (0031460 and 1892914; both Thermo Fisher), and anti-rat (712-036-150; Jackson ImmunoResearch) (all 1:20,000) antibodies, anti-mouse and anti-rabbit conjugated IRDyes (1:5,000, 610-130-121, 610-132-121, and 611-132-122; Rockland), or were replaced by TrueBlot (1:1,000, 18-8816-33 or 18–8817-33; Biomol).

### Bicistronic translation and interferon reporter assay.

HEK293 cells seeded into 96-well plates were transfected the following day with a bicistronic firefly and *Renilla* luciferase reporter construct (40 ng) as well as expression constructs for NSs proteins or the control protein ΔMx (0.1 ng, 1 ng, and 10 ng) via TransIT-LT1. Total transfected DNA was adjusted to equal amounts with the empty vector pI.18. Cells were processed 48 h after transfection, and luciferase activities were determined using the Dual Luciferase Reporter Assay system (Promega) according to the manufacturer’s instructions and a LB 942 TriStar^2^ multimode reader (Berthold Technologies). Means and standard deviations (SDs) were calculated from three technical replicates within each biological replicate.

For interferon reporter assays, cells were seeded and transfected as described above, using p125Luc and pRL-SV40 as reporter plasmids (40 ng each). Luciferase activities were determined 24 h after transfection, and means and SDs were calculated as described above.

### Puromycin labeling.

To monitor ongoing translation, medium was supplemented with 10 ng/ml puromycin and incubated for a further 10 to 30 min at 37°C prior to harvesting (56). Incorporation of puromycin was visualized via immunoblotting as described above.

### Proteomics.

As described previously (19, 27, 51), approximately 2 × 10^8^ HEK293T cells were infected with the recombinant RVFV strain expressing C-terminally TAP-tagged SFSV NSs (rZH548ΔNSs::NSsSFSV-CTAP) at an MOI of 5. The cells were washed with and scraped off in prechilled phosphate-buffered saline (PBS) 16 h postinfection (hpi). The cell pellet was snap-frozen in liquid nitrogen, lysed in TAP buffer (50 mM Tris-HCl [pH 7.5], 100 mM NaCl, 0.2% NP-40, 5% glycerol) supplemented with protease and phosphatase inhibitors, snap-frozen again, and stored at −80°C until further processing. TAP purification was performed by sequential pulldowns using streptavidin-agarose and HA-agarose beads. Bound protein complexes were eventually eluted in Laemmli buffer and subjected to one-dimensional SDS-PAGE prior to trypsin digestion and peptide analysis by liquid chromatography-tandem mass spectrometry (LC-MS/MS), which was described in detail elsewhere (27). Network visualization of high-confidence interactors was generated using STRING database (57).

### Coimmunoprecipitation.

HEK293 cells seeded into 10-cm dishes were transfected with expression plasmids (2 μg each per eIF2B subunit and NSs or control constructs) via the calcium phosphate method (58) and lysed 16 to 24 h after transfection or after additional infection with rZH548ΔNSs::Ren (MOI of 5, 5 hpi). A549 cells were transfected with 4 μg expression plasmids for SFSV NSs or ΔMx using Lipofectamine 2000 (Thermo Fisher). Cells were scraped into PBS and lysed in prechilled lysis buffer (50 mM Tris-HCl [pH 7.0], 150 mM NaCl, 1% IGEPAL-630, 1× protease inhibitor cocktail, 1× phosphatase inhibitor cocktail). Cell debris was removed (10,000 × *g*, 10 min, 4°C), and supernatants were used for further processing. For immunoprecipitation via FLAG or HA, FLAG M2 or HA antibodies were coupled to magnetic beads (143-21D or 10004D; Invitrogen) overnight; for immunoprecipitation of SBP-tagged proteins, streptavidin-coated beads (11205; Invitrogen) were used. Coated beads were then processed according to the manufacturer**’**s recommendations, equilibrated to lysis buffer, and incubated with cell lysate under rotation at 4°C for 4 h or overnight. After extensive washing, bound proteins were eluted by heating in Laemmli gel sample buffer at 94°C for 5 min. For immunoprecipitation via mCitrine, supernatants were applied to prewashed wells of a GFP-multiTrap (Chromotek) and incubated at 4°C for 60 to 90 min. Wells were then washed extensively with lysis buffer, and bound proteins were finally eluted with preheated Laemmli buffer (60°C) under strong agitation for 15 to 20 min.

### Sucrose gradients.

HEK293 cells seeded into 145-mm dishes were either transfected with SFSV NSs or ΔMx expression plasmids (10 μg) or infected the following day with SFSV or rRVFVΔNSs::Katushka (MOI 0.1) or left untreated and harvested 24 h later. Control cells were treated with 1 μM ISRIB (Cay16258-5; Cayman) 1 h prior to harvest. Cells were scraped into PBS and lysed, debris was pelleted (20,000 × *g*, 10 min, 4°C), and 450 μl of the supernatant was loaded onto the gradients. To monitor eIF2B decamerization, 5% to 20% sucrose gradients were poured manually by layering five steps of high-salt lysis buffer [50 mM Tris-HCl (pH 7.4), 400 mM KCl, 4 mM magnesium acetate, 0.5% Triton X-100, 1 mM Tris(2-carboxyethyl)phosphine hydrochloride [TCEP]) supplemented with decreasing sucrose concentrations (5% to 20% sucrose gradient for eIF2B decamerization: 20%, 16.25%, 12.5%, 8.75%, and 5%; 15% to 30% sucrose gradient for eIF2B-phospho-eIF2α association: 15%, 18.75%, 22.5%, 26.25%, and 30%). The gradients were stored at −80°C until usage and allowed to linearize at 4°C overnight prior to loading and ultracentrifugation (SW55, 40,000 rpm, 14 h, 4°C, Beckmann Optima XPN-80). To examine phospho-eIF2αβγ binding to eIF2B, 15% to 30% sucrose gradients were poured in low-salt lysis buffer (50 mM Tris-HCl [pH 7.4], 100 mM KCl, 0.5% Triton X-100, 1 mM TCEP) and subjected to ultracentrifugation (SW55, 45,000 rpm, 6 h 20 min, 4°C). Afterwards, gradients were fractionated manually into 13 or 12 fractions, and aliquots of the crude fractions were finally analyzed by immunoblotting.

### Data availability.

The data sets generated during the present study are available from the corresponding author on reasonable request.
