# Supplementary figures and images for: eIF2B as a Target for Viral Evasion of PKR-Mediated Translation Inhibition
Source: mBio. 2020 Jul 14;11(4):e00976-20. doi: 10.1128/mBio.00976-20 (PMC7360930; doi:10.1128/mBio.00976-20)

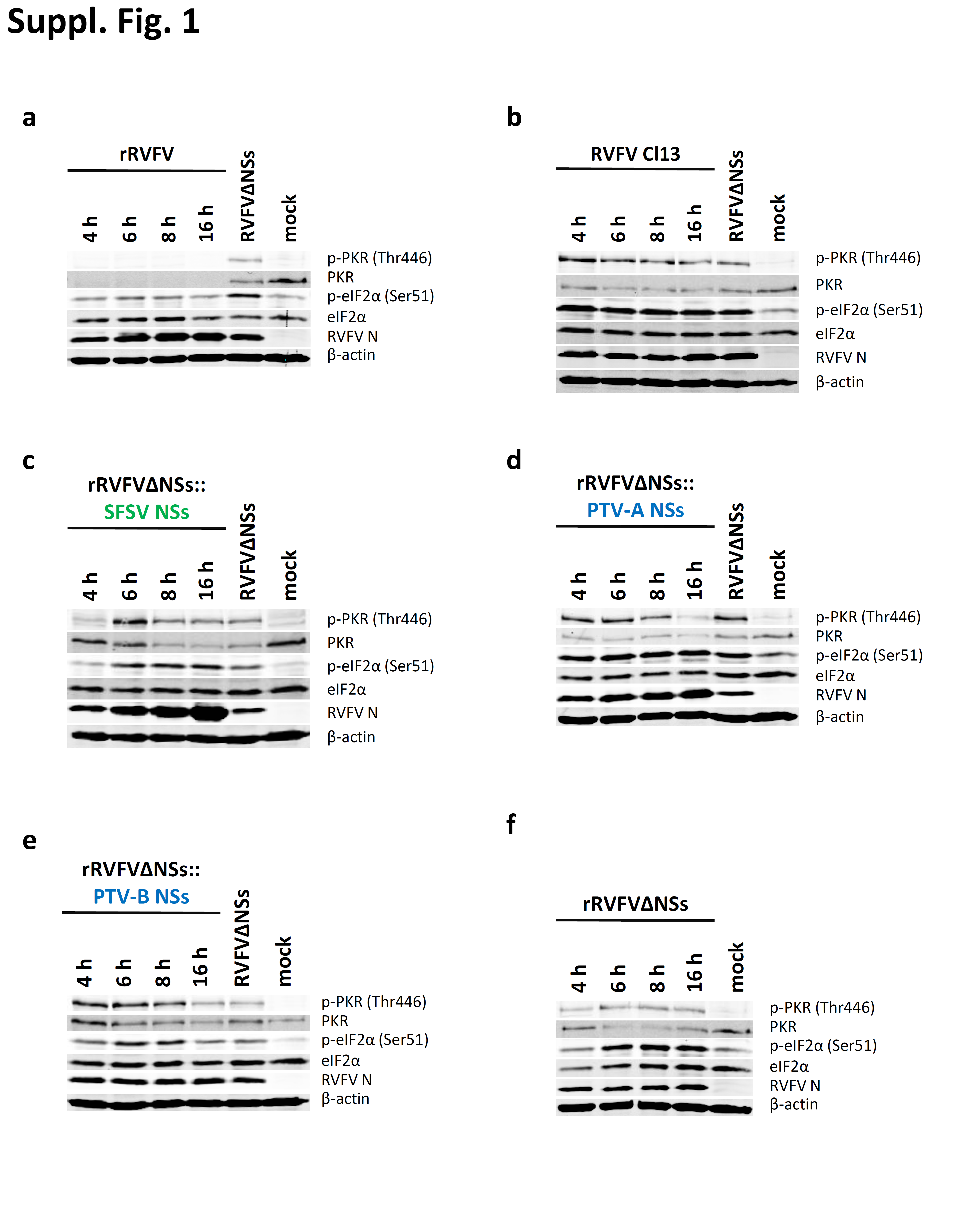

Supplement: FIG S1 [file mBio.00976-20-sf001.tif]

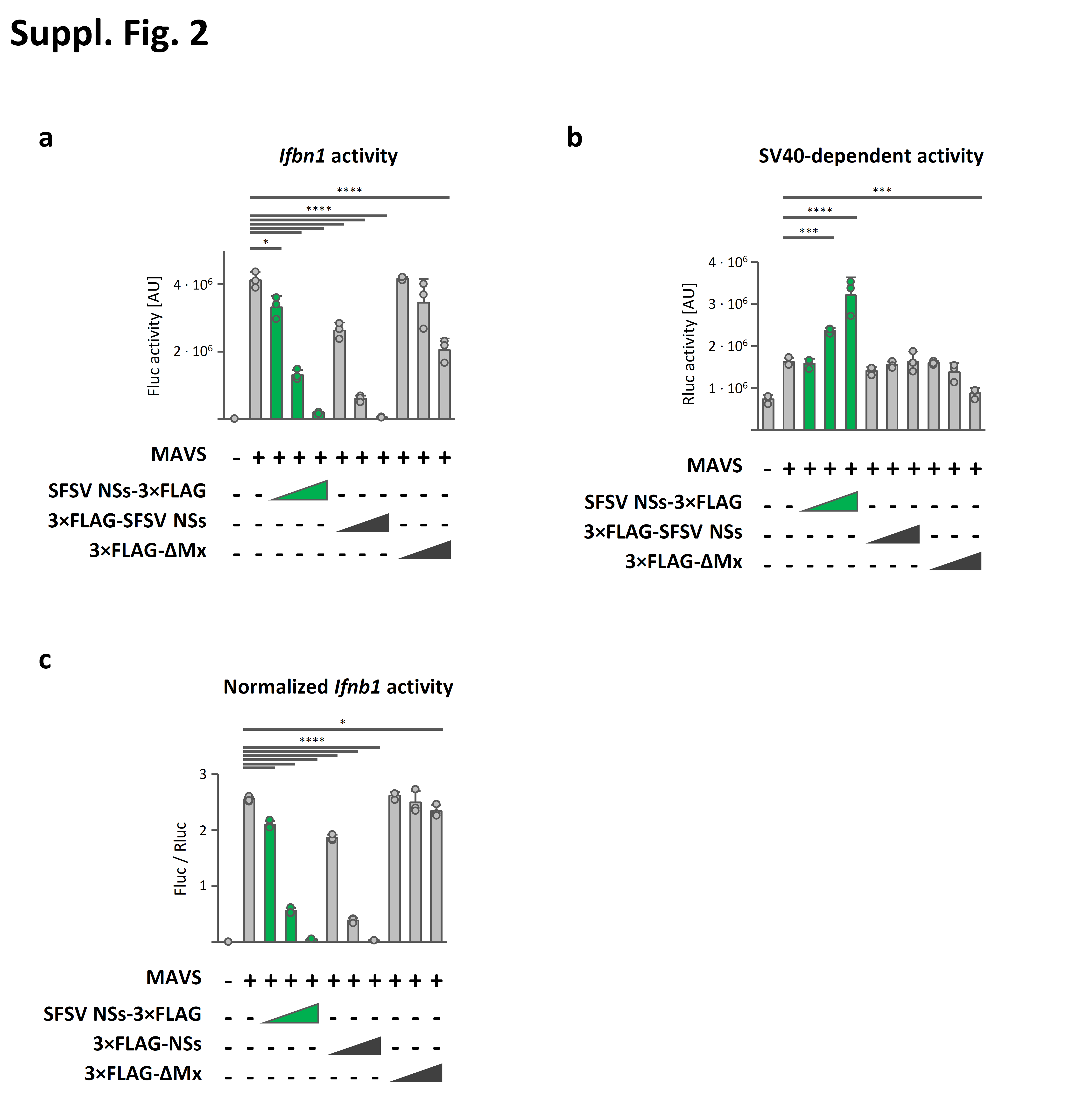

Supplement: FIG S2 [file mBio.00976-20-sf002.tif]

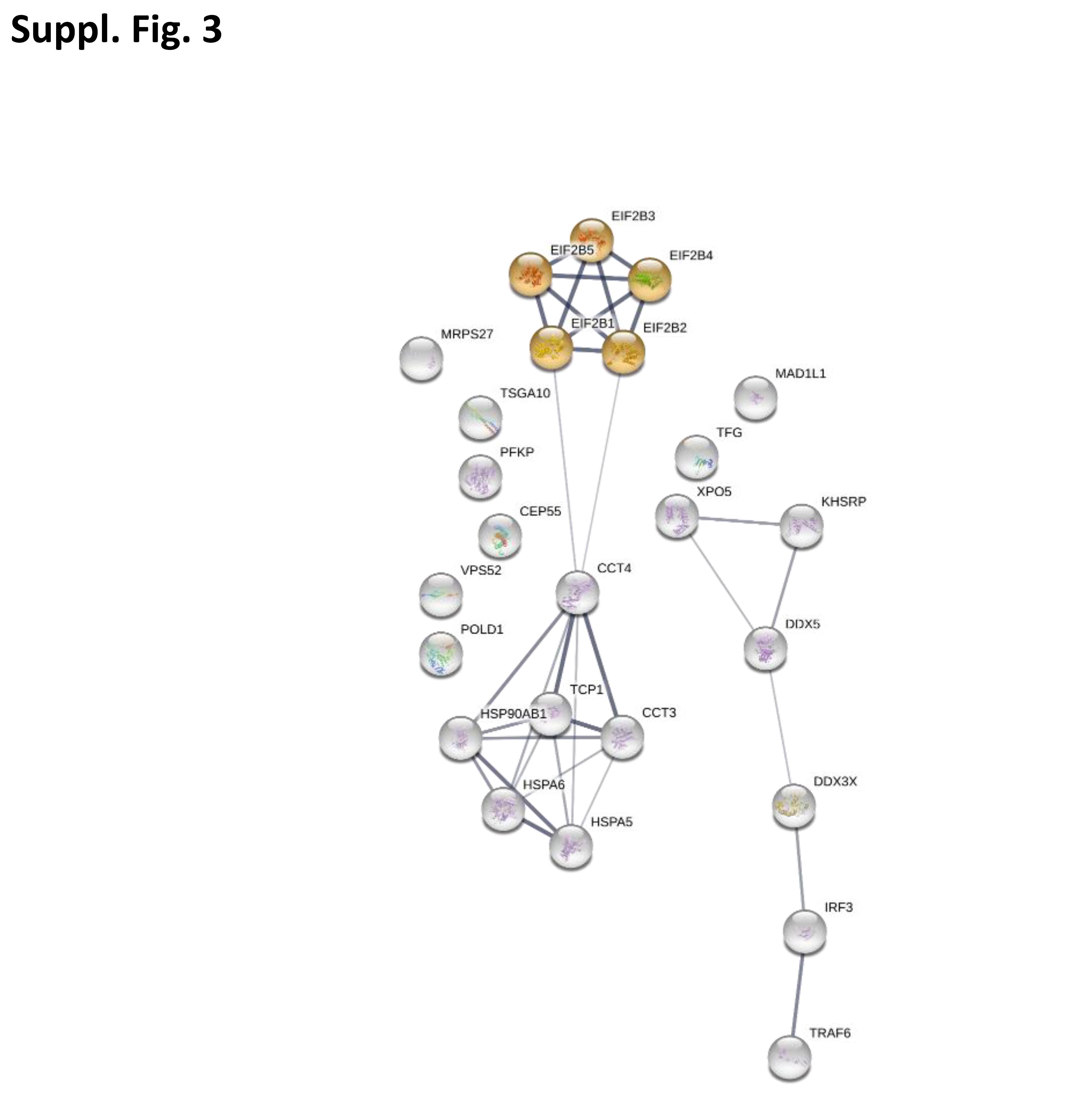

Supplement: FIG S3 [file mBio.00976-20-sf003.tif]

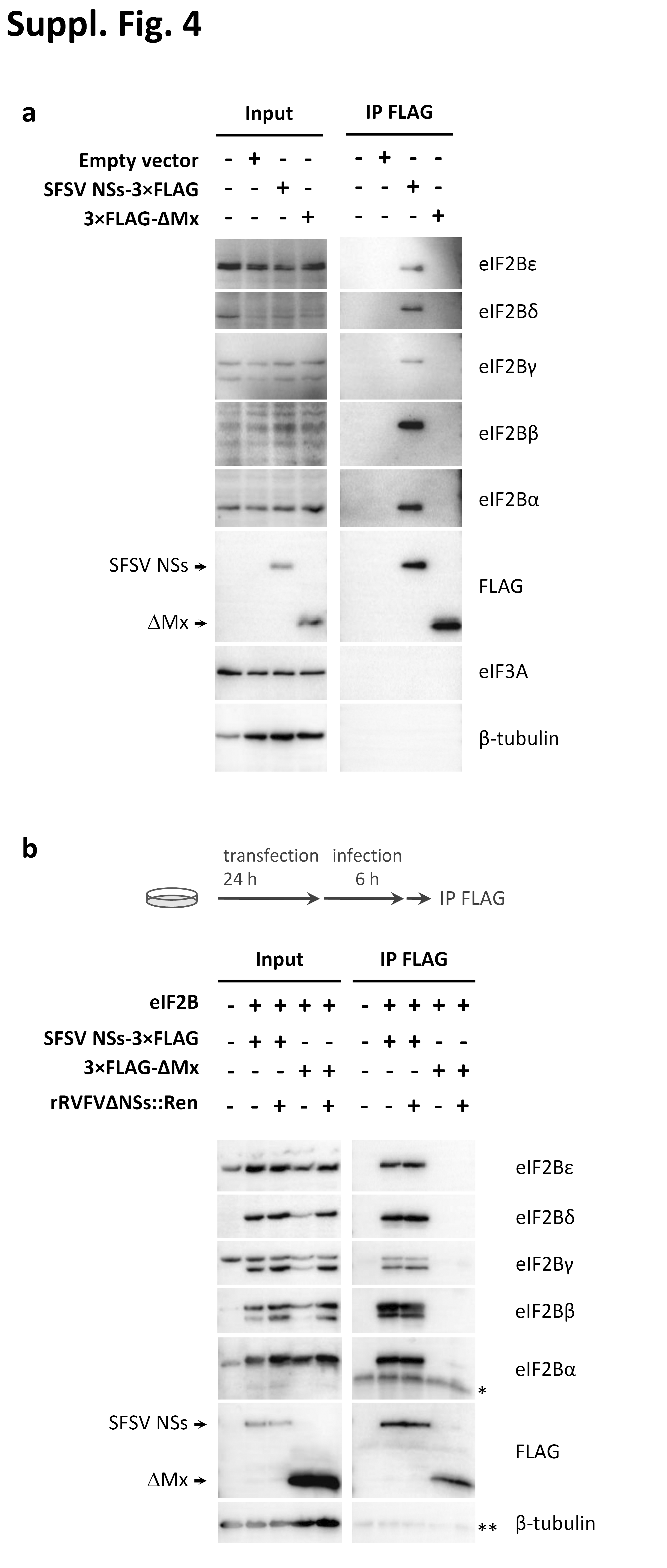

Supplement: FIG S4 [file mBio.00976-20-sf004.tif]

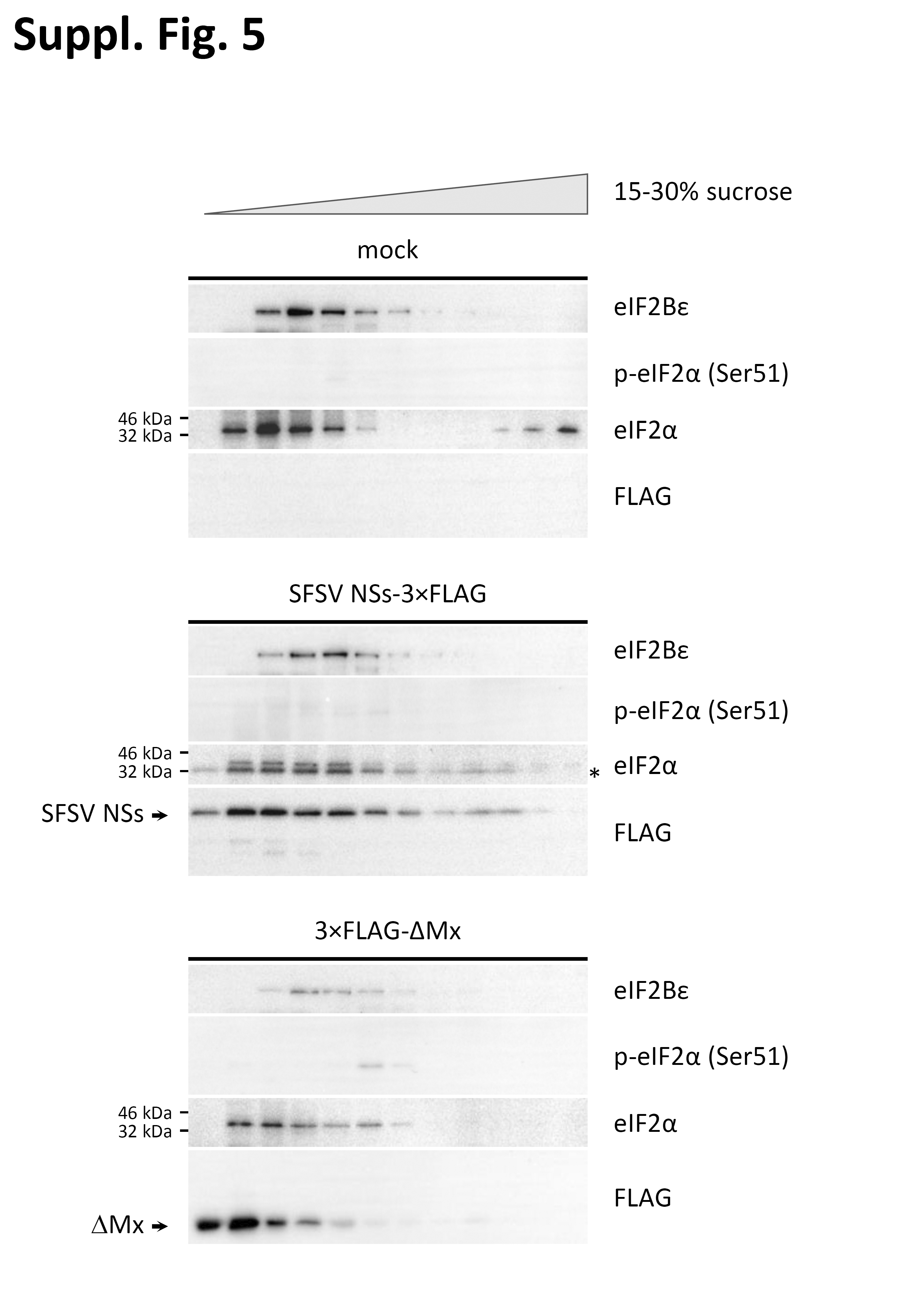

Supplement: FIG S5 [file mBio.00976-20-sf005.tif]
